# Book Reviews

**Published:** 1893-06

**Authors:** 


A PRACTICAL TREATISE ON MATERIA MEDICA AND THERAPEUTICS, WITH ESPECIAL REFERENCE TO THE CLINICAL APPLICATION OF DRUGS, by John V. Shoemaker, A.M., M.D., Professor of Materia Medica, Pharmacology, Therapeutics, and Clinical Medicine, and Clinical Professor of Diseases of the Skin in the Medico-Chirurgical College of Philadelphia ; Physician to the Medico-Chirurgical Hospital ; Member of the American Medical Association of the Pennsylvania and Minnesota State Medical Societies, the American Academy of Medicine, the British Medical Association ; Fellow of the Medical Society of London, etc. Second Edition. Revised. In Two Royal Octavo Volumes. Volume I, 353 pages : Devoted to Pharmacy, General Pharmacology, and Therapeutics and Remedial Agents not Properly Classed with Drugs. Volume II, 680 pages : An Independent Volume upon Drugs. Volume I, in Cloth, $2.50 net ; Sheep, $3.25 net. Volume II, in Cloth, $3.50 net ; Sheep, $4.50 net. Philadelphia : The F. A. Davis Company, Publishers, 1914 and 1916 Cherry Street.

The author has departed from the usual form of works on Materia Medica and Therapeutics, and Vol. I. treats of subjects not found in such works. He believes that for the cure or relief of disease the physician should make use of every means by which the patient may be benefitted. Acting upon this belief, Vol. I. is devoted to the consideration of Pharmacy, Electro-Therapeutics, Massage and Rest Cure, Pneumotherapy, Hydrotherapy, Hypnotism, etc., while Vol. II. is devoted to drugs, their Pharmacology, Physiological Action and Therapy. The book is fully up to date, the author having incorporated in the work and an appendix, many valuable drugs hitherto to be found only in journals and scattered pamphlets.

---

IPECACUANHA AS AN OXYTOCTIC.—Ipecacuanha is said to stimulate uterine contractions; two to three doses of 10 to 15 drops of the wine ipecac, if given every ten minutes, have produced very marked contractions in very short time.—*Der Ærtzl Pract.*

